# IsdB antibody–mediated sepsis following *S*. *aureus* surgical site infection

**DOI:** 10.1172/jci.insight.141164

**Published:** 2020-10-02

**Authors:** Kohei Nishitani, Masahiro Ishikawa, Yugo Morita, Noriaki Yokogawa, Chao Xie, Karen L. de Mesy Bentley, Hiromu Ito, Stephen L. Kates, John L. Daiss, Edward M. Schwarz

**Affiliations:** 1 Center for Musculoskeletal Research, University of Rochester Medical Center, Rochester, New York, USA.; 2 Department of Orthopaedic Surgery, Kyoto University, Kyoto, Japan.; 3 Department of Orthopaedics and; 4 Department of Pathology and Laboratory Medicine, University of Rochester Medical Center, Rochester, New York, USA.; 5 Department of Orthopaedic Surgery, Virginia Commonwealth University Medical Center, Richmond, Virginia, USA.

**Keywords:** Bone Biology, Infectious disease, Bacterial infections, Bacterial vaccines

## Abstract

*Staphylococcus aureus* is prevalent in surgical site infections (SSI) and leads to death in approximately 1% of patients. Phase IIB/III clinical trial results have demonstrated that vaccination against the iron-regulated surface determinant protein B (IsdB) is associated with an increased mortality rate in patients with SSI. Thus, we hypothesized that *S*. *aureus* induces nonneutralizing anti-IsdB antibodies, which facilitate bacterial entry into leukocytes to generate “Trojan horse” leukocytes that disseminate the pathogen. Since hemoglobin (Hb) is the primary target of IsdB, and abundant Hb-haptoglobin (Hb-Hp) complexes in bleeding surgical wounds are normally cleared via CD163-mediated endocytosis by macrophages, we investigated this mechanism in vitro and in vivo. Our results demonstrate that active and passive IsdB immunization of mice renders them susceptible to sepsis following SSI. We also found that a multimolecular complex containing *S*. *aureus* protein A–anti-IsdB–IsdB–Hb-Hp mediates CD163-dependent bacterial internalization of macrophages in vitro. Moreover, IsdB-immunized CD163^–/–^ mice are resistant to sepsis following *S*. *aureus* SSI, as are normal healthy mice given anti-CD163–neutralizing antibodies. These genetic and biologic CD163 deficiencies did not exacerbate local infection. Thus, anti-IsdB antibodies are a risk factor for *S*. *aureus* sepsis following SSI, and disruption of the multimolecular complex and/or CD163 blockade may intervene.

## Abstract 2

Antibodies to iron-regulated surface determinant protein B (IsdB) facilitate bacterial entry into leukocytes and are a risk factor for *S. aureu*s sepsis following surgical site infection.

## Graphical abstract

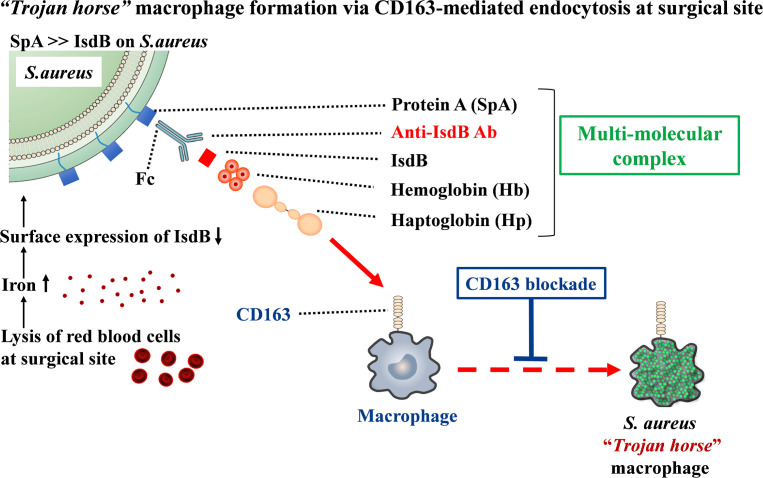

## Introduction

Despite advances in prophylaxis, surgical protocols, and postoperative care, surgical site infections (SSI) remain a serious complication, and the majority are caused by *Staphylococcus aureus* (1). Importantly, over 10% of patients with *S*. *aureus* bacteremia succumb to the infection (2), and mortality after SSI from *S*. *aureus* is about 1% (3). In some regions, over 50% of cases involve methicillin-resistant *S*. *aureus* (MRSA) strains (4), such as the prevalent community-acquired strain USA300 (5). Rigorous intervention studies (e.g., outcomes from the Surgical Care Improvement Project) have demonstrated that infection rates for elective surgery cannot be reduced below 1%–2% (6) and have concluded that unknown host factors are involved (1). As immunization is a cost-effective intervention for the prevention of some infections, there have been major efforts to develop a vaccine against *S*. *aureus*; however, there has been essentially no success in human clinical trials (7). An example of this is the V710 vaccine that was based on active immunization against the iron-regulated surface determinant protein B (IsdB) (8), which is a heme-iron scavenging surface protein that contributes to the pathogenesis of *S*. *aureus* infections in animal models (9, 10). Preclinical development of the V710 vaccine demonstrated that purified IsdB elicits antibodies that block heme-iron scavenging and provide partial protection against *S*. *aureus* bacteremia in animal studies (11). Results from other experiments showed that IsdB-specific antibodies may also promote opsonophagocytosis of *S*. *aureus* (12, 13). However, despite this rigorous preclinical research demonstrating V710 safety and efficacy in mice and rhesus macaques (12, 14), the vaccine was associated with an increased mortality rate from *S*. *aureus* infections among immunized human subjects following elective heart surgery in a phase IIB/III clinical trial (15).

We have also observed adverse outcomes in orthopaedic patients with high titers of circulating antibodies against IsdB at the time of enrollment (16). In our first clinical study, we found that patients with orthopaedic infections who had high titers against IsdB were more likely to die from infections than those who did not have high titers of IsdB (17). Postmortem assessment revealed that patients with MRSA osteomyelitis who succumb to sepsis have “Trojan horse” leukocytes (bacteria infected white blood cells; refs. 18) in their blood and internal organs (19). We also observed high titers of anti-IsdB antibodies in sera and PBMC-cultured medium enriched for newly synthesized anti–*S*. *aureus* antibodies (MENSA) from patients with diabetic foot infections undergoing foot salvage therapy (20). Most recently, we assessed MENSA from 101 patients with musculoskeletal infection (MSKI) (63 culture-confirmed *S*. *aureus*, 38 *S*. *aureus* negative) and 52 healthy controls using machine learning and multivariate receiver operating characteristic curves and found that humoral immunity against IsdB is predictive of active MSKI and MSKI type (21). These MSKI are very challenging to treat, as we found the cure rate of 92 patients with fracture-related infection, 86 patients with prosthetic joint infection, and 49 osteomyelitis to be only 62.1% at 1 year after surgical treatment (22). Given these associations between anti-IsdB antibodies and adverse outcomes following infection, we aimed to elucidate a mechanism by which immunity against IsdB could render patients vulnerable to sepsis and multiorgan failure following *S*. *aureus* SSI that is not caused by preoperative *S*. *aureus* colonization status, diabetes mellitus, obesity, or type of surgical procedure (23).

## Results

**Figure 1:**
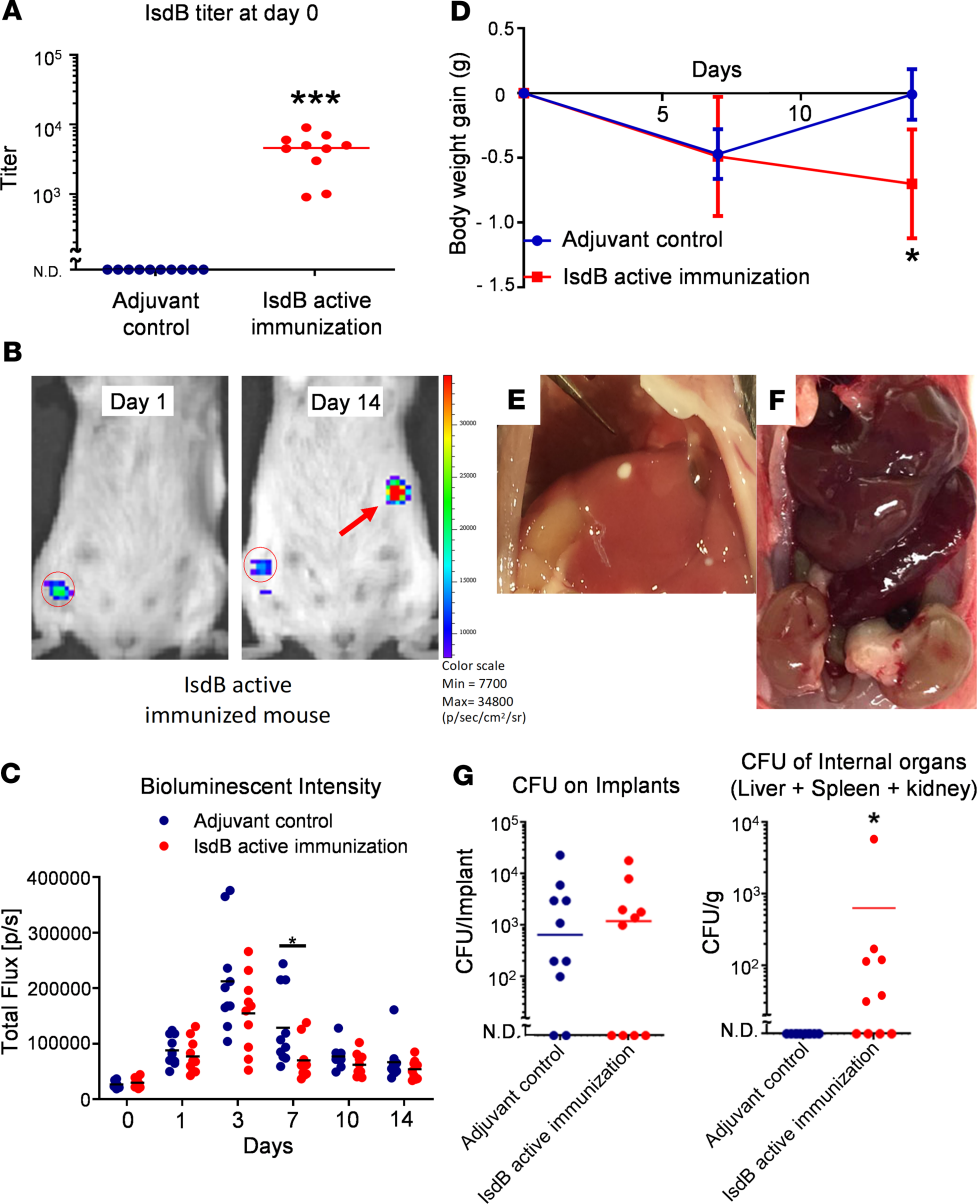
Active IsdB immunization renders mice susceptible to sepsis following SSI. (**A**) Anti-IsdB titers in sera of actively immunized and adjuvant control mice were determined by ELISA before transtibial implant surgery (*n* = 10 per group, ***P < 0.0001 via Mann-Whitney test, lower limit of detection <100). (**B**) Longitudinal BLI images, with heatmap signal intensities, of a representative IsdB actively immunized mouse, with evidence of MRSA dissemination from the surgical site region of interest (ROI, red circled region) to internal organs (red arrow). (**C**) BLI signal within the tibial ROI are shown for individual mice, with the mean for the group (*P < 0.05 on day 7 via exact Wilcoxon test with an adaptive Hochberg multiplicity adjustment). (**D**) The body weight of the mice actively immunized against IsdB protein, or adjuvant only control, was obtained on the indicated days before and after challenge. Note that IsdB-immunized mice did not gain weight after MRSA challenge (*n* = 10 per group, *P < 0.05 on day 14 via 2-way ANOVA). Images of liver abscess (**E**) and pale kidneys (**F**) in anti-IsdB mAb–treated mice. (**G**) CFUs on the tibial pin and in internal organs were determine on day 14 after infection. The incidence and mean level of CFUs on the implants in both groups were similar (lower limit of detection <50). CFUs in internal organs of control mice were not detected (N.D.), while IsdB-immunized mice display evidence of MRSA dissemination (*n* = 10 per group, *P < 0.05 via Fisher’s exact test, lower limit of detection <10).

To test the hypothesis that active vaccination with recombinant IsdB protein (rIsdB) increases *S*. *aureus* dissemination following SSI, immunized mice were challenged with a bioluminescent strain of USA300 via transtibial implantation of a contaminated stainless-steel pin (24). Consistent with prior studies in a murine tail vein sepsis model (14), we found that rIsdB was a potent immunogen, inducing high titers of anti-IsdB IgG antibodies in all immunized mice, and this immunization was protective against the primary infection, as assessed by bioluminescent imaging (BLI) (Figure 1, A and C). However, rIsdB-immunized mice failed to recover their body weight after the septic implant surgery (Figure 1D), and demonstrated disseminated infections, as evidenced by BLI signals and macroscopic abscesses in visceral organs (Figure 1, B and E). While CFU analyses confirmed similar MRSA levels on the implants of all challenged mice, only IsdB-immunized mice had detectible CFU in their internal organs and had macroscopic evidence of kidney damage (Figure 1, F and G). As several groups have published similar murine models of implant-associated *S*. *aureus* infections in which bacterial dissemination could not be detected (25, 26), we found these observations remarkable.

**Figure 2:**
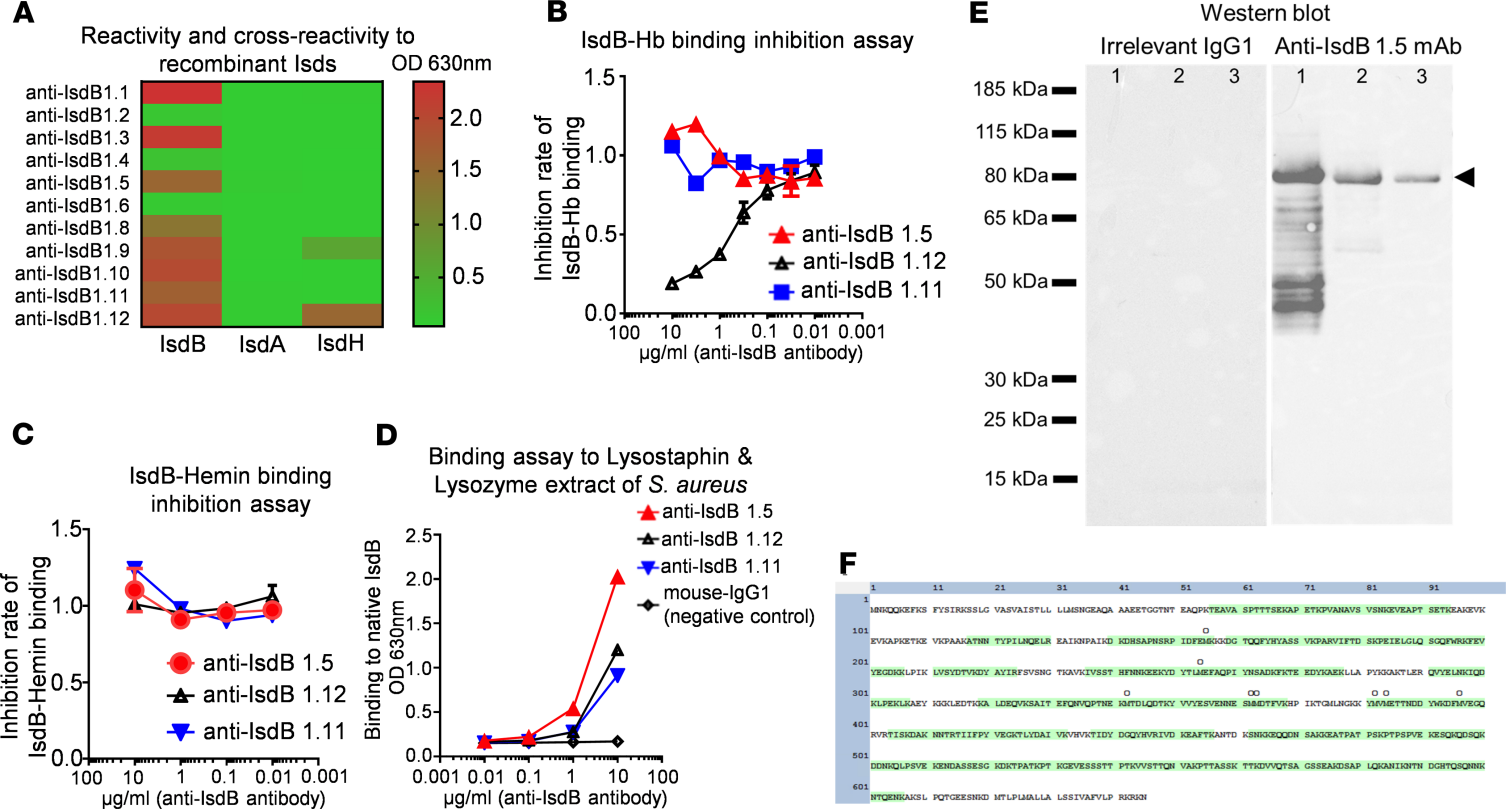
Development of a nonneutralizing anti-IsdB mAb. Mice (*n* = 5) were immunized with recombinant IsdB protein (rIsdB), and their spleen cells were used to make hybridomas, which were screened for anti-IsdB antibodies via ELISA, as described in Methods. Sixteen anti-rIsdB antibody–producing hybridoma pools were obtained for single-cell cloning. (**A**) Twelve anti-IsdB hybridoma cell lines were successfully established and further screened to assess their cross-reactivity with IsdA and IsdH via ELISA. (**B** and **C**) To identify a lead “nonneutralizing” anti-IsdB mAb, which has high avidity to rIsdB without disrupting rIsdB binding to hemoglobin (Hb), we performed sandwich ELISA studies that assessed mAb inhibition of rIsdB binding to Hb and hemin versus an irrelevant anti–*S*. *aureus* amidase (Amd) 1.11 isotype mAb control. Based on these results, anti-IsdB 1.5 was selected as the promising non-neutralizing mAb, whereas anti-IsdB is a 1.12 neutralizing mAb. (**D**) To assess mAb-binding capacity to native IsdB, ELISA was performed with bacterial extract from lysostaphin and lysozyme-digested *S*. *aureus*, which demonstrated dose-dependent binding of all 3 mAbs versus mouse IgG1 negative control. (**E**) Western blot analysis also confirmed specific anti-IsdB 1.5 mAb binding to 83 kDa rIsdB (lane 1) and 80 kDa endogenous IsdB (arrowhead) in *S*. *aureus* lysostaphin and lysozyme extract (lane 2, USA300ΔSpa surface protein extract; lane 3, UAMS-1ΔSpa surface protein extract). (**F**) To further confirm IsdB specificity, lysostaphin and lysozyme protein extract from USA300 ΔSpa was immunoprecipitated with anti-IsdB 1.5 mAb–protein G beads, separated via reducing SDS-PAGE, and the 80 kDa reverse stain band was excised, digested with trypsin, and analyzed by mass spectrometry. The results identified 70 unique peptide sequences (Supplemental Table 1), which cover 70.7% of IsdB (green-labeled sequence; O = oxidized amino acid).

**Figure 3:**
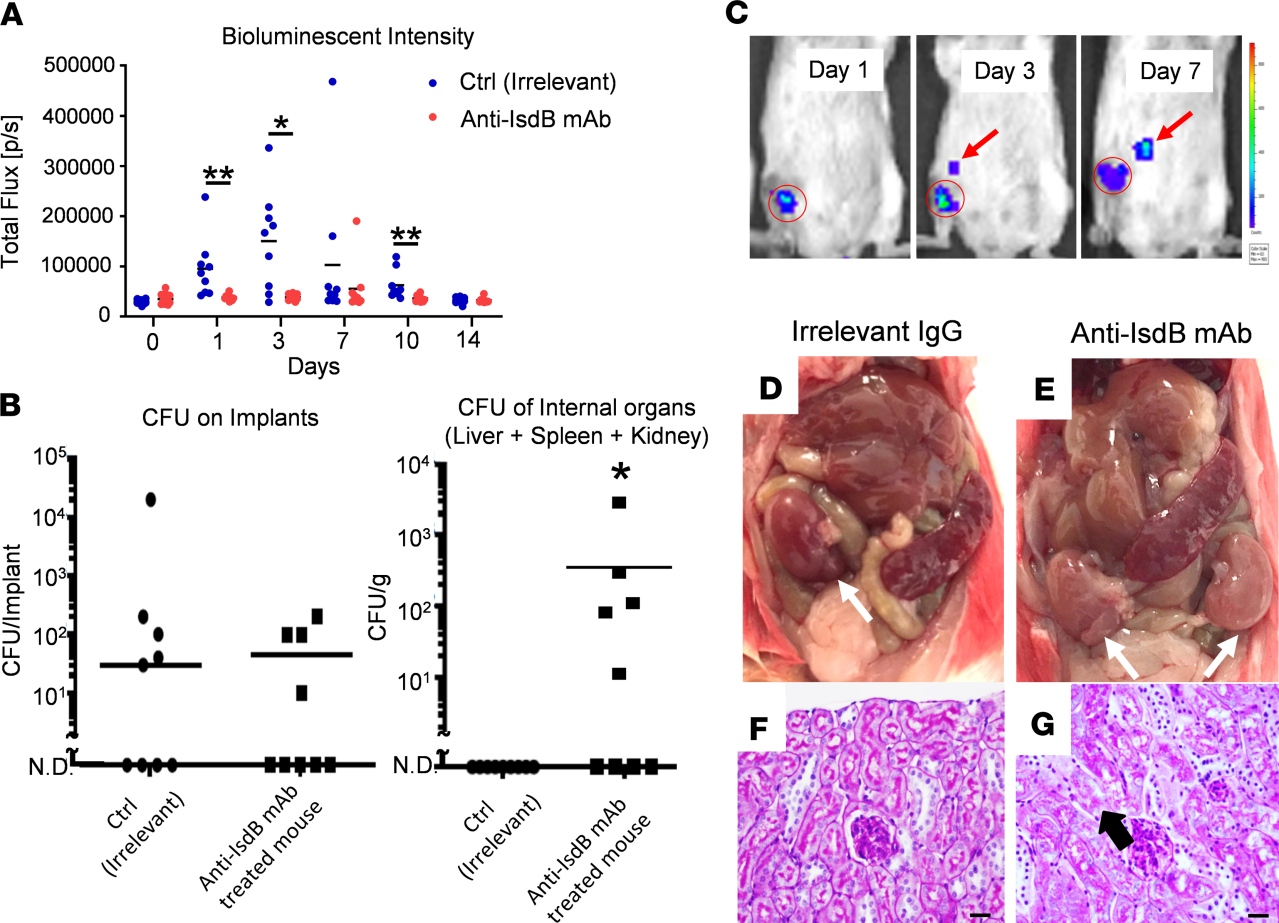
Anti-IsdB mAb passive immunization renders mice susceptible to sepsis following SSI. (**A**) Mice were passively immunized with anti-IsdB or irrelevant control mAb (*n* = 9), challenged with a USA300LAX:Luc-contaminated transtibial implant, and longitudinal BLI was performed as described in Methods. The BLI signal within the tibial ROI for individual mice on the indicated day after challenge is presented with the mean for the group (*P < 0.05 on day 3, **P < 0.01 on days 1 and 10 via exact Wilcoxon test with an adaptive Hochberg multiplicity adjustment). (**B**) CFUs on the tibial pin and in internal organs were determine on day 14 after infection of passively immunized mice. The incidence and mean level of CFUs on the implants in both groups were similar (lower limit of detection <10). However, CFUs in internal organs of control mAb-treated mice were not detected (N.D.), while anti-IsdB mAb–treated mice displayed evidence of MRSA dissemination (*n* = 9, *P < 0.05 via Fisher’s exact test, lower limit of detection <10). (**C**) Longitudinal BLI images with heatmap signal intensities of a representative mouse passively immunized with IsdB with evidence of MRSA dissemination from the surgical site (red circled region) to internal organs (red arrow). (**D**–**G**) Gross anatomy and renal histology of internal organs from mice passively immunized with control IgG and anti-IsdB mAb, illustrating the normal versus pale kidneys (white arrows) and evidence of renal tubular necrosis (black arrow) in anti-IsdB–treated mice. Scale bar: 20 μm.

In order to investigate the direct effects of anti-IsdB humoral immunity on MRSA dissemination following SSI, we generated anti-IsdB mAbs and assessed their ability to block rIsdB binding to hemoglobin (Hb) in vitro (Figure 2). This work produced a hybridoma clone (1.5) that secretes high-affinity nonneutralizing anti-IsdB mAb, which specifically binds to IsdB without cross-reactivity to other *S*. *aureus* proteins and does not interfere with IsdB binding to Hb. We then used this anti-IsdB mAb, and an irrelevant mAb control, to passively immunize mice before challenge with a MRSA-contaminated transtibial pin (Figure 3). Similar to active immunization with rIsdB, challenged mice that received the anti-IsdB mAb displayed a significant decrease in BLI at the surgical site on days 1, 3, and 10 after infection compared with placebo-treated mice (Figure 3A) but suffered from bacterial dissemination to internal organs (Figure 3, B and C) and ischemic kidneys, with histopathological evidence of renal tubular necrosis (Figure 3, D–G).

**Figure 4:**
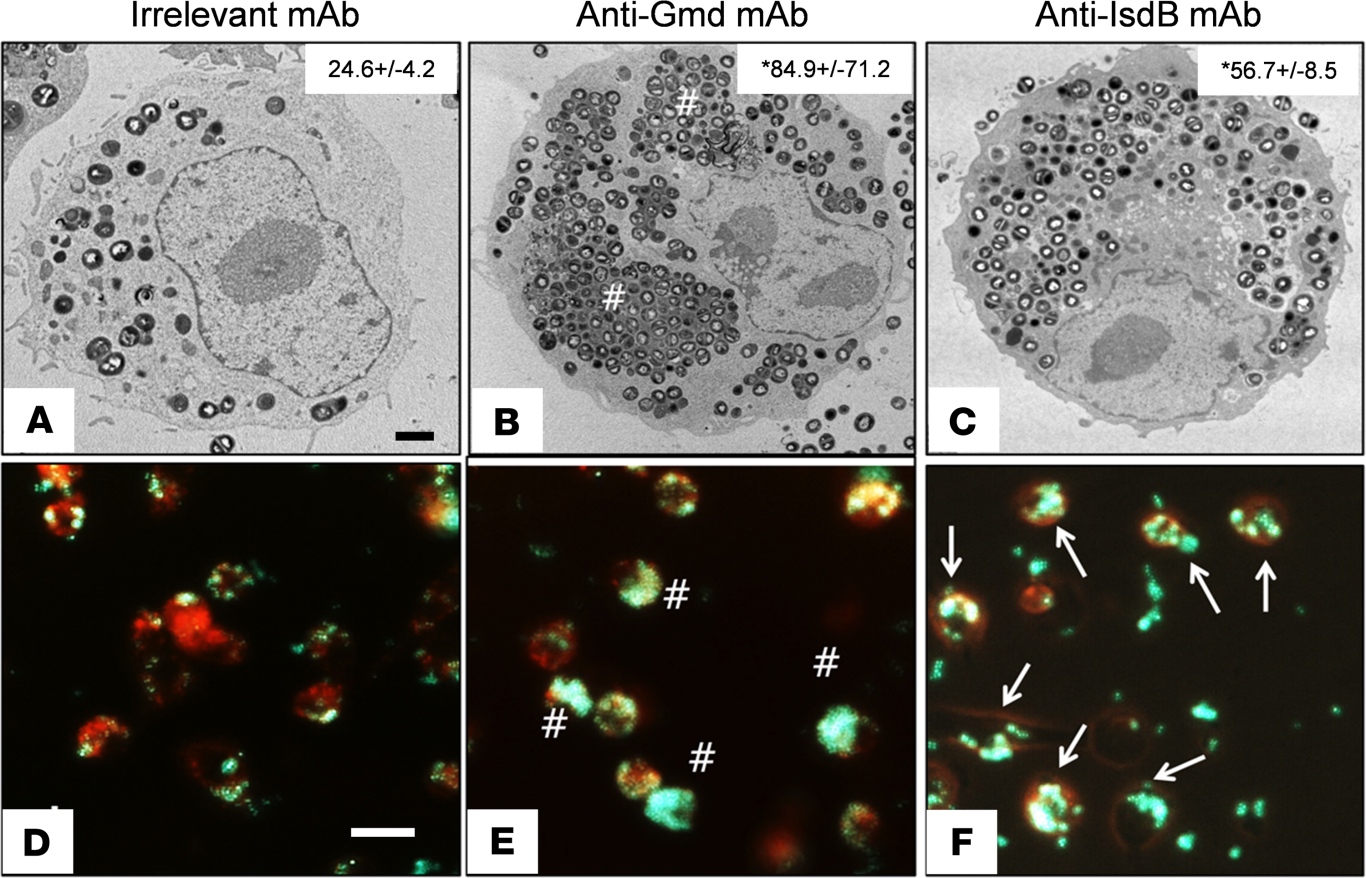
Anti-IsdB mAb induces increased *S.* *aureus* internalization by macrophages. RAW 264.7 cells grown in serum-containing media were challenged with MRSA (USA300LAC) treated with 50 μg/mL(**A**) irrelevant IgG (negative control), (**B**) anti-Gmd (1C11 positive control), or (**C**) anti-IsdB, for 2 hours at (MOI = 10), before TEM as described in Methods. Representative images (original magnification, ×5000) are shown with quantification of the number of bacteria per cell (mean ± SD, *n* = 4, *P < 0.05 vs. IgG control via Kruskal-Wallis test; scale bar: 2 μm.). This experiment was repeated with LysoTracker Red–labeled RAW cells challenged with GFP+ UAMS-1 via real-time fluorescent microscopy (original magnification, ×100) (**D**–**F**)**.** Note the megaclusters (pound signs indicates clustered GFP signal in **E**) and Trojan horse macrophages (arrows indicate punctate GFP signal in **F**). Scale bar: 20 μm.

The leading theory to explain the dissemination of *S*. *aureus* from SSI to internal organs is the so-called Trojan horse leukocyte hypothesis (18). This theory posits that intracellular infection of macrophages and/or neutrophils at the surgical site allows for bacterial replication and translocation to internal organs in an immune-privileged environment. To directly test anti-IsdB mAb effects on macrophage internalization of *S*. *aureus*, we performed in vitro transmission electron microscopy (TEM) and fluorescent microscopy studies with RAW264.7 cells (Figure 4). These studies showed that anti-IsdB mAb markedly increased bacterial uptake versus an IgG1 mAb of unrelated specificity. However, in contrast to an anti-glucosaminidase mAb known to protect mice from implant-associated osteomyelitis via opsonophagocytosis of large *S*. *aureus* clusters (27, 28), the bacteria internalized by RAW cells exposed to anti-IsdB mAb were not clustered and did not appear to be within vacuoles. Other than the increased number of bacteria per cell, these infected RAW cells appeared similar to infected RAW cells exposed to the irrelevant mAb control and resembled the so-called Trojan horse macrophages that are hypothesized to disseminate bacteria following SSI (18) and are present in patients who succumb to *S*. *aureus* sepsis (19).

**Figure 5:**
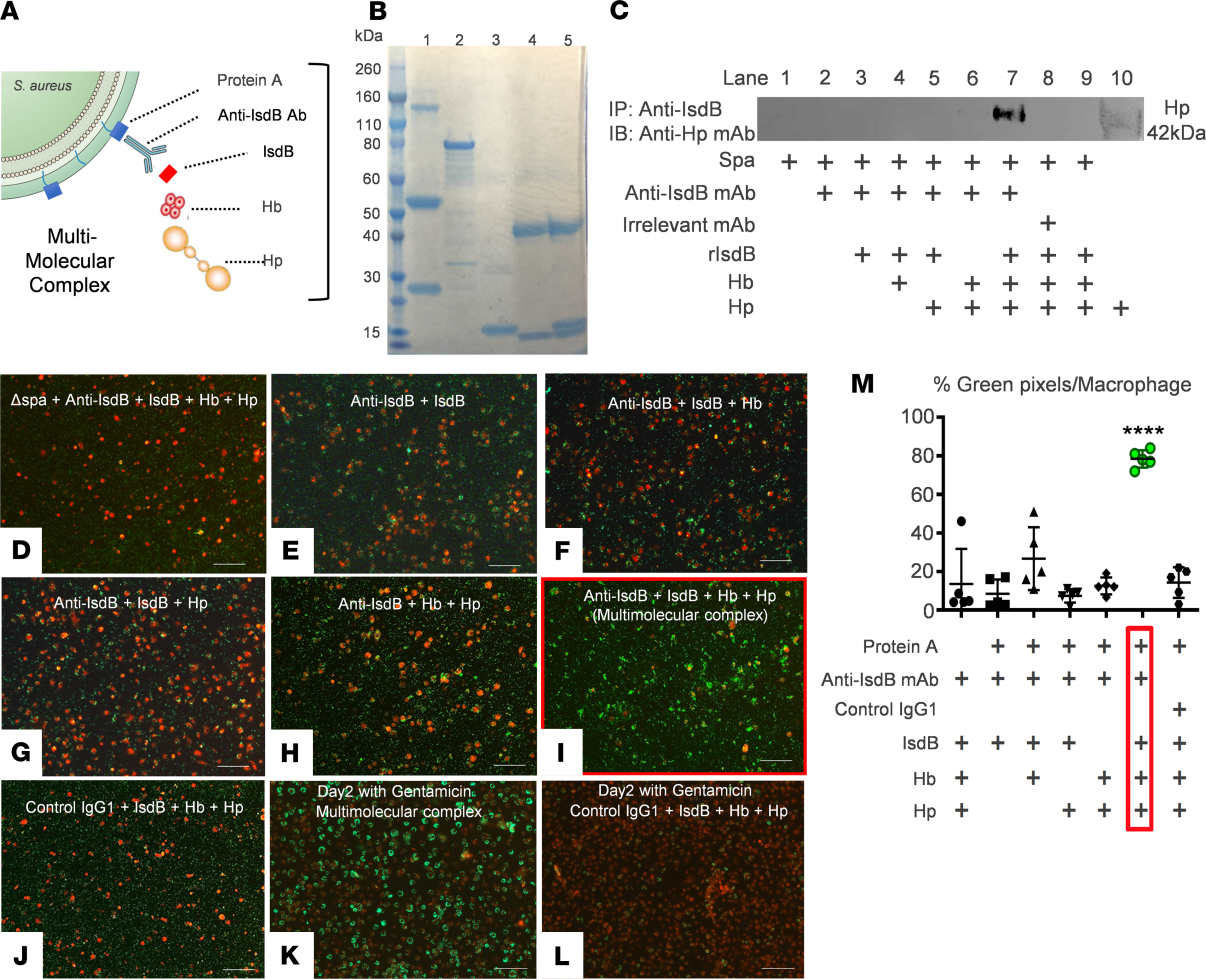
All components of the multimolecular complex physically associate and are required for anti-IsdB antibody–mediated *S.* *aureus* internalization of macrophages. (**A**) A schematic illustration of the 5 proteins in the multimolecular complex with their hypothesized orientation is shown. In this model, anti-IsdB antibody attaches to the bacterial surface via Spa-Fc binding and to soluble IsdB via Fab binding. Antibody-bound IsdB protein also binds to Hb-Hp, such that all 5 proteins are physically associated in the multimolecular complex. (**B**) Immunoprecipitation-Western blotting was performed to demonstrate specific protein interactions. Coomassie blue–stained SDS-PAGE is shown to illustrate the purity of the input proteins. Lane 1, anti-IsdB mAb; lane 2, recombinant IsdB; lane 3, hemoglobin (Hb); lane 4, haptoglobin (Hp); and lane, 5, Hb-Hp complex. (**C**) For immunoprecipitation, a combination of anti-IsdB mAb or irrelevant IgG control mAb (50 μg/mL), recombinant IsdB (40 μg/mL), Hb (25 μg/mL), and Hp (35 μg/mL) were incubated with *S*. *aureus* protein A–coupled (Spa-coupled) beads. Eluates were assessed for Hp content via Western blot with anti-Hp antibody. Note that Hp detection in this immunoprecipitation-Western assay requires a multimolecular complex that includes Spa, anti-IsdB mAb, IsdB, and the Hb-Hp complex, as omission of any of these proteins results in the loss of detection. In vitro *S*. *aureus* internalization assays were performed to quantify GFP^+^ *S*. *aureus* (UAMS-1) in LysoTracker Red–stained primary bone marrow–derived macrophages incubated with the indicated multimolecular complex proteins. Representative fluorescent micrographs were obtained 3 hours later (original magnification, ×10) (**D**–**J**) or after 48 hours culture in gentamicin (scale bar: 100 μm) (**K** and **L**). (**M**) Quantification of bacterial internalization was performed via Visiopharm (Supplemental Figure 1), and the data are presented with the mean ± SD. In the protein A–negative group, the UAMS-1ΔSpa strain was used instead of UAMS-1 to show that Spa is required for multimolecular complex–mediated bacterial endocytosis (*n* = 5, ****P < 0.0001 vs. all other groups via 1-way ANOVA with post hoc Tukey’s test).

Considering that Hb is the primary target of IsdB (29), and that Hb-haptoglobin (Hb-Hp) complexes are cleared via CD163-medicated endocytosis by macrophages (30), we explored the possibility that anti-IsdB mAb–mediated infection of macrophages via CD163 receptor–mediated endocytosis is facilitated by a multimolecular complex containing Spa–anti-IsdB antibody–IsdB-Hb-Hp (Figure 5A). To test this, we evaluated the physical association of these proteins in vitro. Immunoprecipitation studies with purified proteins demonstrated that all of the components of this multimolecular complex are required to physically link Spa to Hp (Figure 5, B and C). Moreover, we found that all of these protein components are required for efficient *S*. *aureus* uptake by primary bone marrow–derived macrophages and RAW cells grown in serum-free media that does not contain Hb-Hp (Figure 5, D–M; and Supplemental Figures 1 and 2; supplemental material available online with this article; https://doi.org/10.1172/jci.insight.141164DS1).

**Figure 6:**
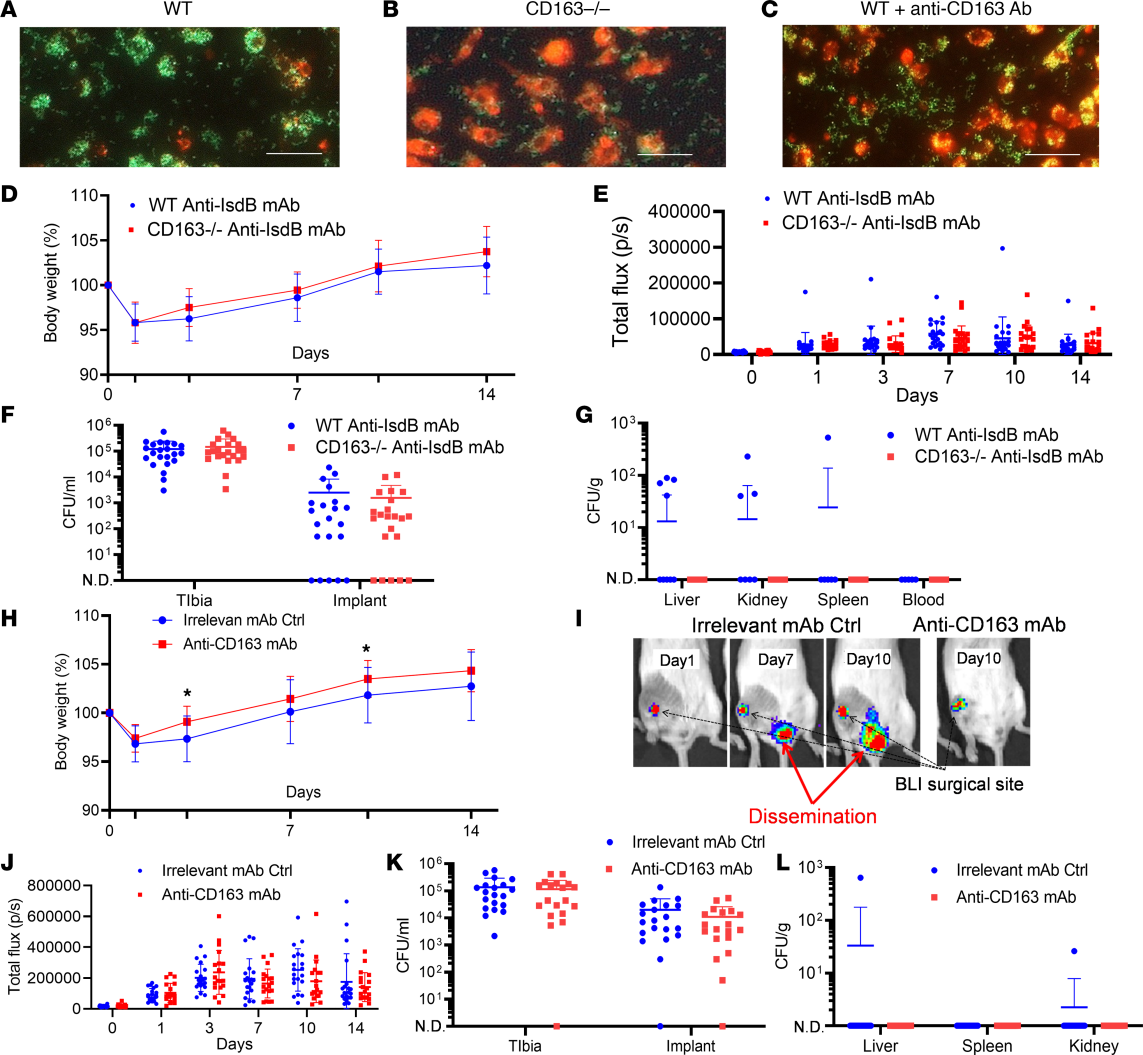
CD163 is required for multimolecular complex internalization of *S.* *aureus* by macrophages in vitro and dissemination following SSI in vivo. Assessment of in vitro *S*. *aureus* infection of primary bone marrow–derived macrophages cultured with all components of the multimolecular complex was performed as described in the legend for Figure 5. Representative fluorescent images of GFP^+^ UAMS-1 in cultures of LysoTracker Red–labeled (**A**) WT macrophages, (**B**) CD163^–/–^ macrophages, and (**C**) WT macrophages pretreated with anti-CD163 antibodies (original magnification, ×100; scale bar: 10 μm). (**D**) WT (C57BL/6) and CD163^–/–^ mice in a C57BL/6 background were passively immunized with anti-IsdB and challenged with a MRSA-contaminated transtibial implant, as described in the legend for Figure 1. Total body weight over the 14-day infection period is presented as the fraction (%) of mice per group (*n* = 22). (**E**) Longitudinal BLI, with local (**F**) and systemic (**G**) CFU data per mouse (mean ± SD) per group are presented. (**H**) WT BALB/c mice were passively immunized with anti-IsdB mAb, as described in the legend for Figure 3, and treated with anti-CD163 mAb or irrelevant IgG before challenge with a MRSA-contaminated transtibial implant (*n* = 20). The percentage body weight change over the 14-day infection period is presented (mean ± SD) per group (*P < 0.05 on days 3, 10, and 14 via 2-way ANOVA). (**I**) BLI images are shown to illustrate the USA300LAC:lux dissemination in an irrelevant mAb control mouse, while no MRSA dissemination was detected in any of the mice treated with anti-CD163 mAb, as illustrated by the representative BLI image. (**J**) Longitudinal BLI, with local (**K**) and systemic (**L**) CFU data per mouse (mean ± SD) for each group are presented (lower limit of detection <50 [**F** and **K**]; <100 [**G** and **L**]).

To evaluate the role of CD163 in *S*. *aureus* uptake by macrophages in vitro and MRSA dissemination following SSI in vivo, we used mice deficient for CD163 (CD163^–/–^ mice) and a potentially novel neutralizing anti-CD163 mAb (Supplemental Figure 3). The results showed that multimolecular complex–mediated internalization of GFP^+^ UAMS-1 is lost in cultures of CD163^–/–^ macrophages and in cultures of WT macrophages pretreated with anti-CD163 Ab (Figure 6, A–C). In vivo, CD163^–/–^ mice or WT mice in a C57BL/6 background passively immunized with anti-IsdB mAb challenged with a MRSA-contaminated transtibial implant demonstrated a faster recovery of total body weight compared with control WT mice (Figure 6D). While both groups of mice passively immunized with anti-IsdB mAb demonstrated similar local infections (Figure 6, E and F), MRSA dissemination to internal organs was only detected in the WT mice (Figure 6G). Consistently, WT mice passively immunized with anti-IsdB mAb treated with anti-CD163 mAb before challenge with a MRSA-contaminated transtibial implant also demonstrated significantly improved total body weight recovery postoperatively (Figure 6H). They also demonstrated similar local infections (Figure 6, J and K) and were completely protected from MRSA dissemination (Figure 6, I and L).

## Discussion

Although elective surgeries are for the most part very safe, SSI remain a major clinical problem for the rare patients that contract them. In the case of prosthetic joint infections, infection rates, the primary pathogen, treatment algorithm, and prevalence of poor outcomes have not changed since the original revision surgery standards of care were established half a century ago (1, 31, 32). There is also expert consensus that local treatments, including antibiotic-loaded bone cement, are not effective to treat chronic MSKI (33) and that development of an effective immunotherapy against *S*. *aureus* is among the highest priorities in orthopaedics (1). Unfortunately, all active and passive vaccine trials to date have failed (34). It is noteworthy that these trials were based on safety and efficacy studies in small animals, and antibody opsonophagocytic activity was the primary biomarker of immunity in human volunteers and patients. In retrospect, the inability of opsonophagocytic antibodies to demonstrate efficacy in patients is not surprising since people with agammaglobulinemia show no increase in the incidence of *S*. *aureus* infection (34). Moreover, utilization of standard rodent models has not been predictive of patient responses to Staphylococcal infections for either protective efficacy (35, 36) or human inflammatory responses to sepsis (37).

To the end of an effective immunization against *S*. *aureus*, we have pursued nontraditional preclinical and clinical research strategies. Our vaccine discovery approach has focused on murine models with quantitative outcomes, including in vivo planktonic growth; bacterial biofilm on the implants; Staphylococcus abscess communities; invasion and colonization of the osteocytic-canalicular network of cortical bone; osteolysis; and implant osseointegration (24, 27, 38–41). While this work has identified the autolysin antigens as potential targets (27, 41–43), it also identified IsdB as the most immunodominant antigen (17). Our clinical research approach has been focused on elucidating the immune proteome against *S*. *aureus* in patients with MSKI and correlating their humoral immunity with their clinical outcome (16, 17, 19–21, 44–46). While this work also found autolysin antigens to have human vaccine potential, we also found a clear signal that humoral immunity against IsdB is associated with poor clinical outcomes, including amputation and septic death (16, 17, 20). In these findings, together with the results of the V710 vaccine phase IIB/III clinical trial, which demonstrated increases death in IsdB-immunized patients from *S*. *aureus* infections (15), we aimed to elucidate the mechanism responsible.

**Figure 7:**
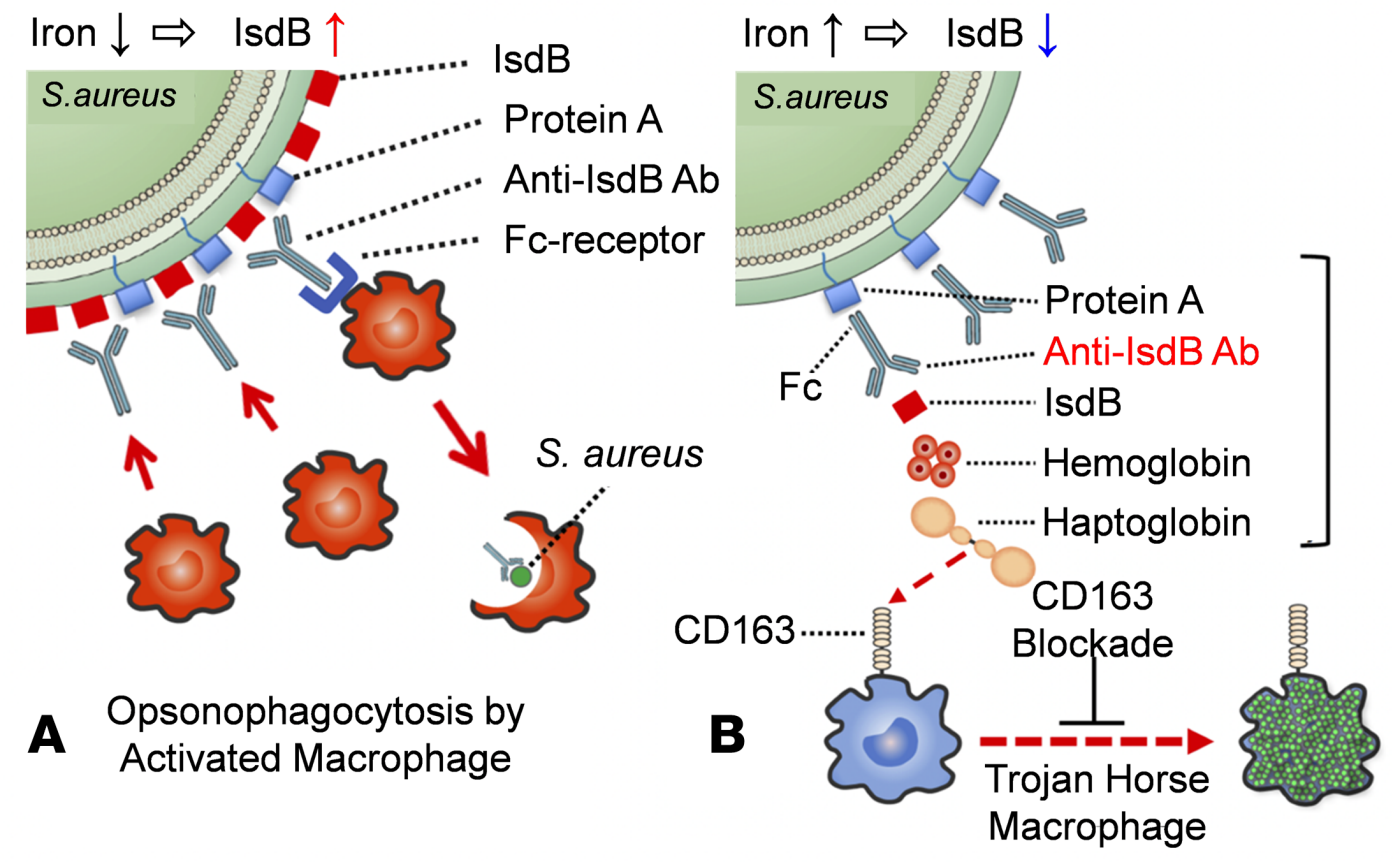
Schematic model of protective versus pathogenic anti-IsdB antibody activity during *S. aureus* hematogenous versus surgical site infection. (**A**) During hematogenous infection, *S*. *aureus* responds to the low level of nutritional iron by inducing IsdB production to levels that greatly exceed Spa expression levels, such that anti-IsdB antibody–Fab binding to its antigen on the bacterium is favored. This leads to host protection via opsonophagocytosis by activated macrophages and effective clearance of the infection. (**B**) In contrast, the high levels of iron at surgical sites that result from bleeding and lysis of red blood cells stimulates the downregulation of IsdB by *S*. *aureus*, such that anti-IsdB antibody–Fc binding to Spa is favored over Fab-antigen binding. Subsequent binding by the few shed IsdB molecules to the Fab of the nonneutralizing anti-IsdB antibody and Hb-Hp complex bind to the opsonized IsdB, establishing a multimolecular complex (bracket) that facilitates *S*. *aureus* internalization of scavenger macrophages via CD163 receptor–mediated endocytosis. These leukocytes infected with proliferating bacteria act as Trojan horse macrophages to disseminate the *S*. *aureus* throughout the host. Based on this theory, drugs that disrupt multimolecular complex formation and/or biological CD163 blockage are predicted to inhibit Trojan horse macrophage formation and prevent sepsis.

Here, we show that an anti-IsdB mAb can facilitate *S*. *aureus* internalization and survival in macrophages in vitro and mediate *S*. *aureus* dissemination in a murine model of implant-associated osteomyelitis via a multimolecular complex that includes Spa, the anti-IsdB mAb, IsdB, Hb-Hp, and CD163. While these data establish a pathogenic mechanism by which *S*. *aureus* exploits anti-IsdB immunity to infect host leukocytes and cause disseminated infections, they are also in apparent conflict with preclinical studies demonstrating that IsdB immunization protects mice from sepsis (11, 14). To reconcile this, we propose distinct models of host immune responses against IsdB in the settings of hematogenous sepsis versus SSI (Figure 7). In the case of hematogenous infections, free Fe^++^ levels are low and *S*. *aureus* IsdB surface expression levels are high to address this nutritional requirement. This renders the bacteria highly susceptible to anti-IsdB antibody–Fc receptor–mediated opsonophagocytosis and clearance by activated neutrophils and macrophages. In contrast, Fe^++^ levels at surgical sites are high due to bleeding and subsequent red blood cell lysis. Thus, IsdB expression is downregulated to levels markedly below Spa on the bacterial surface, such that nonantigen binding of anti-IsdB antibodies to *S*. *aureus* via the Fc-domain is favored. This model also posits that limited amounts of IsdB are still expressed and shed from the bacterial surface in the high Fe^++^ SSI environment, such that anti-IsdB antibodies bind this limited soluble IsdB via Fab-antigen binding. Importantly, evidence that low levels of IsdB are shed from bacteria comes from our in vitro findings in which rIsdB was omitted from *S*. *aureus* internalization assays, and a low level of internalization was still observed (Figure 5, H and M). Since IsdB binds to Hb, this opsonized IsdB binds to the Hb-Hp complex, which then binds to CD163 on macrophages and potentially neutrophils that also express CD163 (47–49). This prohibits Fc receptor opsonophagocytosis by leukocytes and allows entry and colonization of scavenger macrophages and neutrophils via CD163-mediated endocytosis. Our data also suggest that disruption of multimolecular complex formation and/or CD163 blockade may be approaches to prevent sepsis following SSI. Additionally, as the absence of a predictive small animal model for *S*. *aureus* immunization research has been identified as a major limitation for clinical translation (36), our finding that murine host responses to IsdB in an orthopaedic SSI model are fundamentally different from results in a well-established sepsis model (11, 14) highlights the importance of face validity in preclinical research.

There are several limitations to our study that should be noted. The first is that while our reductionist model with an anti-IsdB mAb has been useful to ask specific questions about its function in vitro and in vivo, the human humoral response against *S*. *aureus* infection and IsdB vaccination is polyclonal, and thus anti-IsdB mAb 1.5 cannot reflect the actual response in patients. Second, it should be noted that our theory of anti-IsdB antibody–mediated Trojan horse leukocyte formation during SSI is a preliminary model that warrants further investigations. Additional focused studies include experiments to establish that (a) Fc receptor–mediated opsonophagocytosis dominates in host environments where Fe^++^ is limited, (b) IsdB surface expression is downregulated on bacteria at the site of infection, and (c) Spa-deficient strains have reduced bacterial dissemination in SSI models. Finally, we did not formally investigate immunosuppression in CD163^–/–^ mice or mice treated with anti-CD163. Thus, although we did not observe any changes in the course of *S*. *aureus* infection in IsdB-immunized and unimmunized mice, this topic remains open for future investigations and validation of CD163 blockade as a potential intervention.

## Methods

### Bacterial strains

Methicillin-sensitive *S*. *aureus* (UAMS-1) and MRSA (USA300) were used for in vitro experiments as previously described (27). For in vitro experiments that aimed to assess the role of *S*. *aureus* protein A (Spa), a UAMS-1 strain genetically deficient for Spa was generated via phage exchange. A bioluminescent strain of USA300 (USA300LAC:lux) (50) was used for all in vivo challenge experiments.

### In vivo studies

All in vivo challenge experiments used the murine transtibial pin model of implant-associated osteomyelitis with 6- to 8-week-old female BALB/c or C57BL/6 mice purchased from The Jackson Laboratory, as we have previously described (24, 27, 38, 41). For experiments with CD163-deficient mice, CD163^–/–^ breeder mice on a C57BL/6 background were obtained from The Jackson Laboratory (Cd163^tm1.1(KOMP)Vlcg^).

### Active immunizations

Active immunizations were performed as we have previously described (42). Briefly, rIsdB (GenScript) was emulsified with adjuvant (Sigma Adjuvant System, S6322, MilliporeSigma) and i.p. injected 3 times (day –28, –14, and –7) to immunize the mice. Adjuvant was injected as the same manner as a control. Serum antibody titers against the antigen were determined by ELISA.

### Generation of mAb-producing hybridomas and validation of mAbs

#### Nonneutralizing anti-IsdB 1.5 mAb.

rIsdB was used to immunize mice, screen hybridoma pools, and clone anti-IsdB antibody–producing hybridoma cell lines as described in the legend for Figure 2. Based on these results, anti-IsdB 1.5 mAb was chosen as a nonneutralizing antibody that binds to native IsdB without inhibiting IsdB binding to the Hb-Hp complex. It also does not cross-react with any other Isd proteins. The anti-IsdB 1.5 mAb–producing hybridoma cell line has been deposited into a cell line bank for distribution (ATCC, catalog SD-7579).

#### Neutralizing anti-CD163 3B5-5 mAb.

Recombinant protein containing the CD163 scavenger receptor cysteine-rich domains 2–4 and flanking His and AVI tags was generated by GenScript. This antigen used to immunize mice and generation of anti-CD163 mAb–producing hybridoma cell lines is described in the legend for Supplement Figure 3. The results identified a clear lead neutralizing anti-CD163 mAb (3B5-5), which has been deposited into a cell line bank for distribution (ATCC, catalog SD-7580).

### Passive immunizations

Passive immunization of mice with anti-IsdB mAb 1.5 or irrelevant IgG (mouse IgG1 isotype control from MOPC-21 murine myeloma cell line, MilliporeSigma), and CD163 blockade with mAb 3B5-5, were performed as we have previously described (42). Briefly, mAb were purified from hybridoma culture supernatants via protein G column chromatography, and mice received a single injection (40 mg/kg/i.p.) the day before septic transtibial pin implant surgery.

In vitro *S*. *aureus* uptake assays with RAW cells and primary bone marrow–derived macrophages

TEM was performed on RAW 264.7 cells (ATCC) as we have previously described (27). Primary macrophages were labeled with LysoTracker Red (Thermo Fisher Scientific) in the presence of Hb-Hp (25 μg/mL and 35 μg/mL, respectively, both from MilliporeSigma) complex. Some macrophages were preincubated with neutralizing anti-CD163 mAb (anti-CD163 3B5-5, 50 μg/mL) or anti-CD163 polyclonal antibody (Proteintech) before adding Hb-Hp complex. GFP^+^ *S*. *aureus* (UAMS-1) was incubated with 40 μg/mL rIsdB and 50 μg/mL irrelevant IgG (MilliporeSigma) or anti-IsdB mAb at MOI = 50 or 100 for 3 hours. Some of the macrophages were further cultured for 3 hours in the presence of 100 μg/mL gentamicin (Thermo Fisher Scientific) and 20 μg/mL lysostaphin (MilliporeSigma) or for 48 hours in the presence of 100 μg/mL gentamicin before fluorescence microscopy at ×100x and quantification as described in the legend for Supplemental Figure 2.

### In vitro multimolecular complex assays

Anti-IsdB mAb (anti-IsdB 1.5), rIsdB (GenScript), Hb (MilliporeSigma), and Hp (MilliporeSigma) was separated by SDS-PAGE under reducing conditions, followed by Coomassie blue staining to illustrate the purity of the input proteins. For immunoprecipitation, a combination of rIsdB (40 μg/mL), Hb (25 μg/mL), and Hp (35 μg/mL) was incubated at 37°C for 2 hours. They were immunoprecipitated with anti-IsdB mAb (anti-IsdB 1.5, 50 μg/mL) or irrelevant IgG (50 μg/mL, MilliporeSigma) and *S*pa-coupled beads (Thermo Fisher Scientific). Proteins bound to the beads were analyzed via immunoblotting with anti-Hp mAb (Abcam).

### Statistics

We used Mann-Whitney test, exact Wilcoxon test with an adaptive Hochberg multiplicity adjustment, 2-way ANOVA, Fisher’s exact test, and 1-way ANOVA with post hoc Tukey’s test to assess significance. A *P* value of less than 0.05 was considered significant.

### Study approval

All animal experiments conducted at the University of Rochester were approved by the University of Rochester Committee for Animal Resources.

## Author contributions

KN, MI, YM, and NY produced the mAbs and executed most of the experiments shown in Figures 1–6 and Supplemental Figures 1–3, with contributions from CX, KLDMB, and JLD. HI, SLK, and EMS contributed to writing the manuscript. HI, SLK, JLD, and EMS designed the study, supervised, and coordinated the execution of the study.

## Supplementary Material

Supplemental data

## Version 1 10/02/2020

Electronic publication
