# Supplementary material for: IsdB antibody–mediated sepsis following S. aureus surgical site infection
Source: JCI Insight. 2020 Oct 2;5(19):e141164. doi: 10.1172/jci.insight.141164 (PMC7566716; doi:10.1172/jci.insight.141164)
Supplement: Supplemental data [file jciinsight-5-141164-s191.pdf]

Supplemental Figure 1

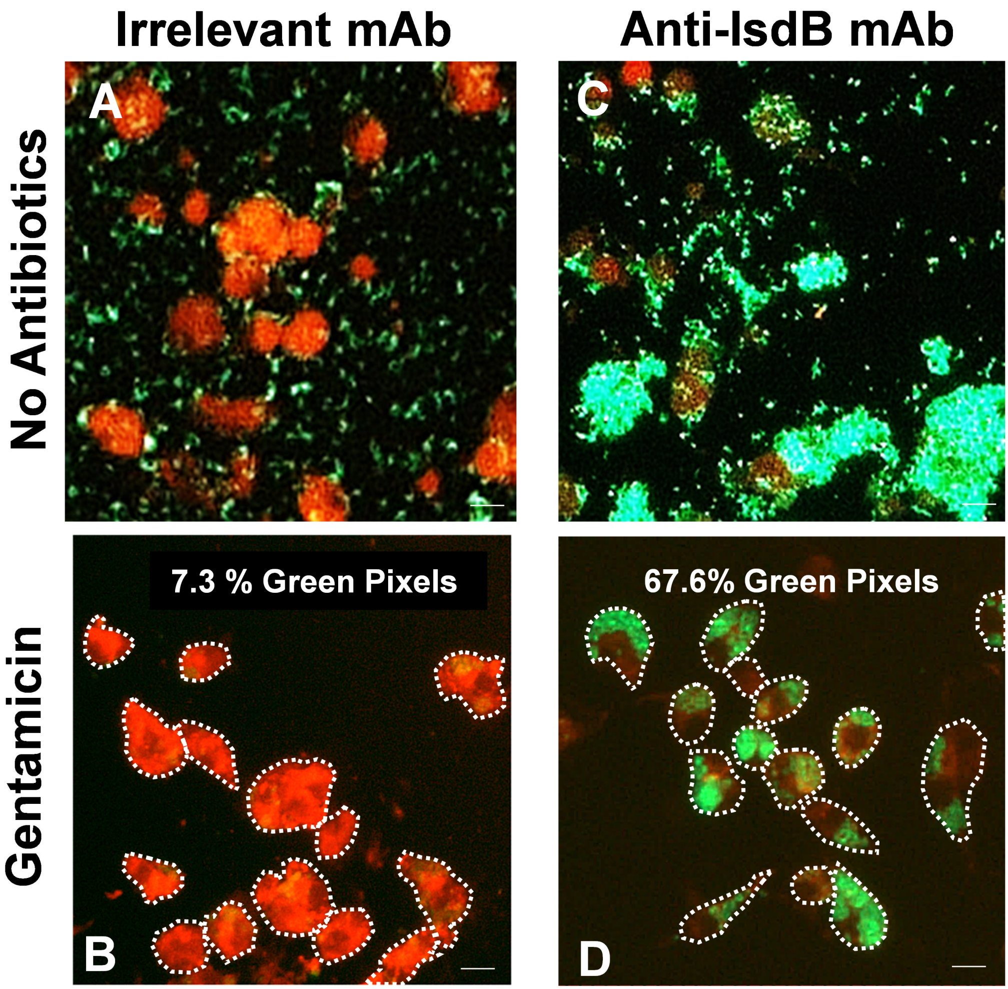

Supplemental Figure 1. Quantification of intracellular *S. aureus* in macrophages. Murine macrophages were cultured in serum free media without Hb-Hp, then labeled with LysoTracker Red in the presence of Hb-Hp complex, and exposed to GFP+ UAMS-1 in the presence of recombinant IsdB, and irrelevant IgG (**A** & **B**) or anti-IsdB mAb (**C** & **D**) for 3hr. Some of the cultures were further incubated for 3hrs in the presence of and lysostaphin prior to fluorescent microscopy at 100x. The digital images were then processed gentamicin Visiopharm to assess the relative amount of intracellular bacteria as follows. First, macrophage area without bacteria was segmented in the red channel, and bacteria area within the macrophage area was segmented in the green channel. The green area smaller than 100 pixels was deleted as floating bacteria outside macrophages. Then the % of green pixels within macrophage area (dotted lines in **B** & **D**) was determined. Note the prevalence of extracellular bacteria in the absence of anti-IsdB mAb (**A**) that are cleared by gentamicin (**B**), and the anti-IsdB mAb induced intracellular GFP+ clusters (**C**) that persist following gentamicin treatment (**D**). Scale bar: 10μm.

Supplemental Figure 2

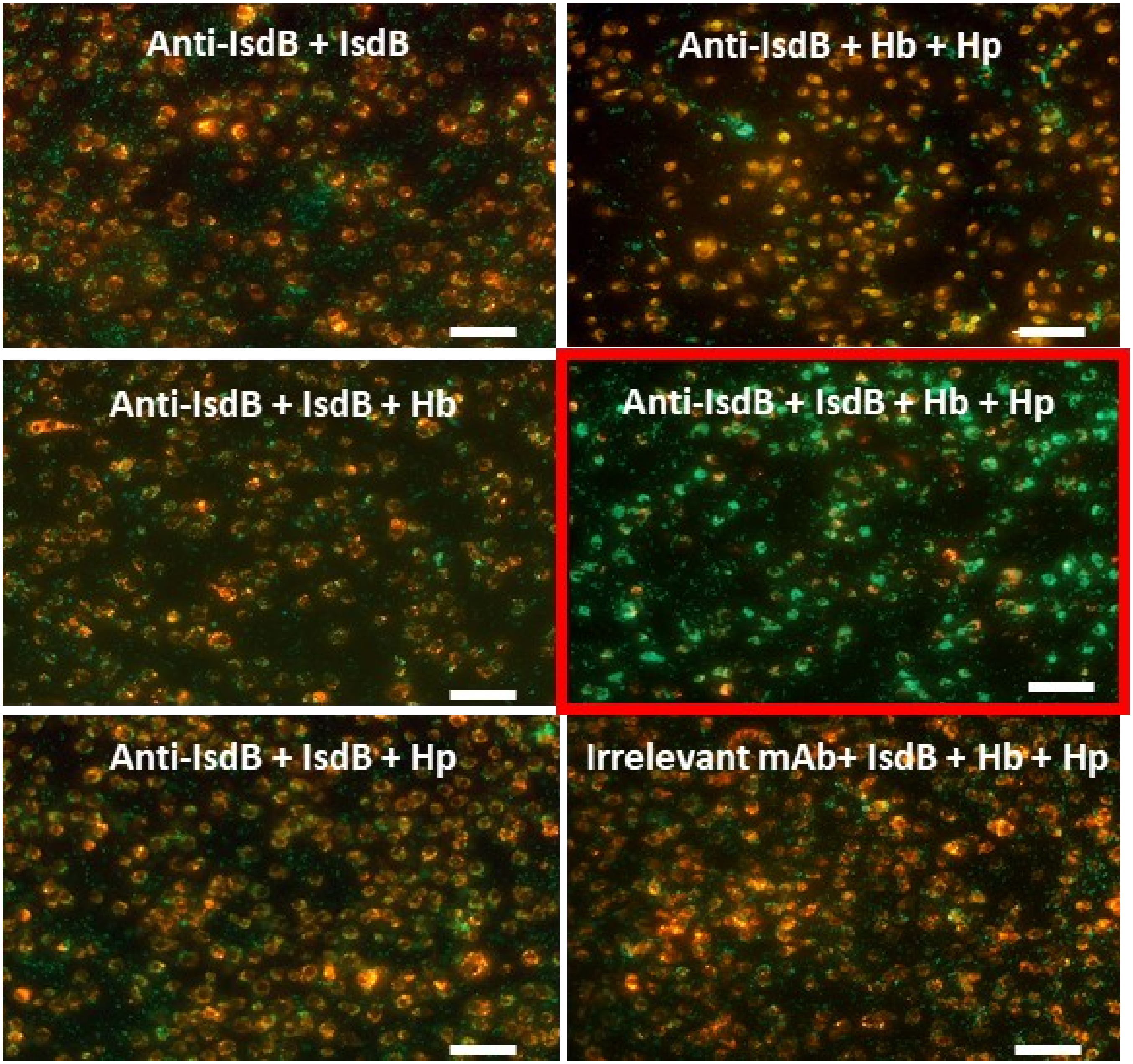

Supplemental Figure 2. All components of the multimolecular complex are required for efficient internalization of *S. aureus* in RAW cells. Assessment of in vitro *S. aureus* infection of RAW cells was performed as described in Figure 3, and representative fluorescent images of LysoTracker Red labeled macrophages and GFP+ UAMS-1 are shown at 10x (Scale bar: 100μm). Note that the efficient *S. aureus* uptake by RAW cells only occurs when all components of the multimolecular complex are present (red box).

Supplemental Figure 3

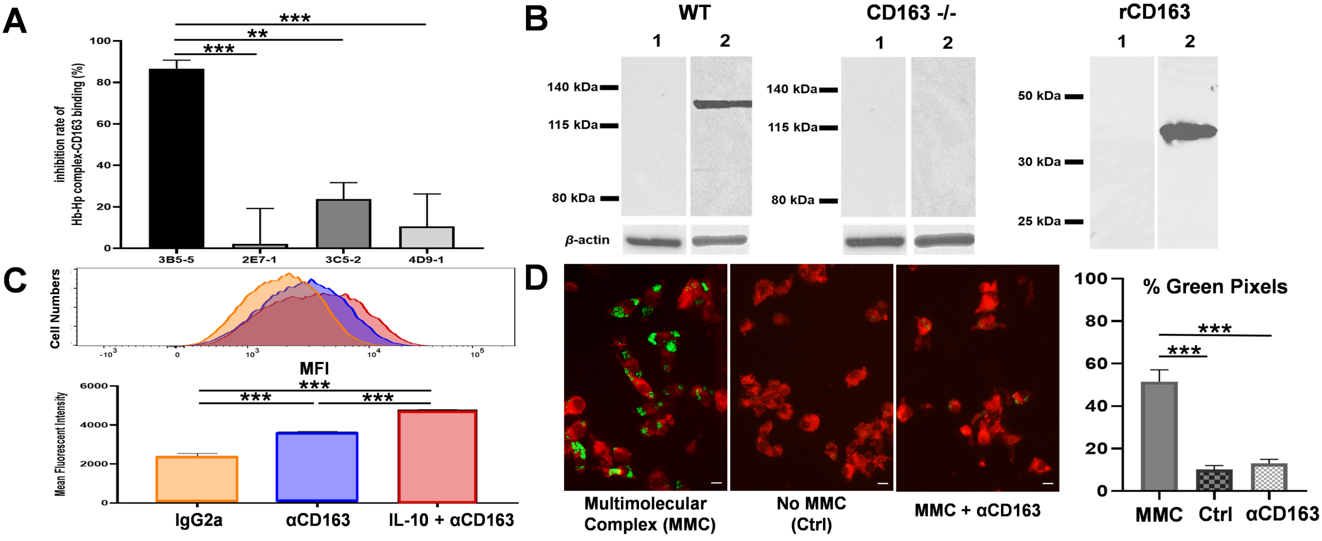

Supplemental Figure 3. Development of neutralizing anti-CD163 monoclonal antibody. His-tagged recombinant protein containing the cysteine-rich domains 2-4 of the human CD163 scavenger receptor (rCD163) was purified from *E. coli* (GeneScript Inc., Piscataway, NJ, USA). Following confirmation of the molecular weight of rCD163 (38kDa) via SDS-PAGE and Western blotting with an anti-His antibody (data not shown), the rCD163 was used to immunize mice for mAb generation as we described for anti-IsdB mAb generation (Supplemental Fig. 1). **A**, Four anti-CD163 antibody producing hybridomas were cloned via limited dilution, and their anti-CD163 mAbs were purified and screened for inhibition of Hb-Hp complex binding to rCD163 using a sandwich ELISA. Inhibition was calculated as (OD^{630}(rCD163 only) – OD^{630}(rCD163 + 50ug/ml of each anti-CD163 monoclonal antibody) / OD^{630}(rCD163 only) $\times$ 100 (%). The results showed that anti-CD163 mAb 3B5-5 had the greatest neutralizing activity (n = 3, **p < 0.01 and ***p < 0.001 via one-way ANOVA with post-hoc Tukey test). **B**, Western blotting under reducing conditions was performed to confirm mAb 3B5-5 binding (Lane 2) to native CD163 in total protein liver extract from WT and CD163-/- mice. Western blotting with an anti-β actin antibody (Novus Biologicals, Centennial, CO, USA) was performed to control for protein loading and extract integrity, together with irrelevant mouse IgG controls (Lane 1, irrelevant mouse IgG (Sigma-Aldrich, St Louis, MO, USA) was used as first antibody), and the rCD163 protein control. **C**, Flow cytometry with murine bone marrow derived macrophages cultured without or with IL-10 (50ng/ml Peprotech, Rocky Hill, NJ, USA) for 24 hours was performed with anti-CD163 3B5-5 mAb conjugated with Alexa Flour 647, or mouse isotype IgG2a conjugated with Alexa Fluor 647. Representative histograms from four independent experiments are shown, with the mean +/- SD of the mean fluorescent intensity (MFI). Note the 1.5-fold increase in MFI without IL-10, and 2.2-fold increase in MFI with IL-10 stimulation (n = 3, ***p < 0.0001, one-way ANOVA with post-hoc Tukey's test). **D**, Anti-CD163 3B5-5 mAb inhibition of *S. aureus* internalization by RAW cells was performed as described in Supplemental Fig. 2. Representative 100X fluorescent images of UAMS-1 GFP+ infected RAW cells cultured in gentamicin are shown with % green pixel quantification (n = 4, mean +/- SD, ***p < 0.0001 one-way ANOVA with post-hoc Tukey test). Scale bar: 10μm. The 3B5-5 anti-CD163 mAb producing hybridoma cell line has been deposited (ATCC, Manassas, VA, USA, Cat# SD-7580).

Supplemental Table 1

Confidence	Sequence	Modifications	# PSMs	DeltaM [ppm]	Percolator q-Value	Percolator PEP	Xcorr
High	YVVYESVENNESMMDTFVK	2xOxidation [M13; M14]	2	-2.25	2.04E-03	4.69E-07	6.97
High	TEAVASPTTTSEKAPETKPVANAVSVSNKEVEAPTSETK		3	-0.04	2.04E-03	1.94E-04	6.39
High	YVVYESVENNESMMDTFVK	1xOxidation [M13; M14]	3	0.46	2.04E-03	6.35E-05	6.28
High	YVVYESVENNESMMDTFVK		1	0.91	2.04E-03	2.01E-04	5.71
High	DVVQTSAGSSEAKDSAPLQK		2	-1.79	2.04E-03	1.17E-05	5.52
High	APETKPVANAVSVSNKEVEAPTSETK		3	-0.37	2.04E-03	1.50E-06	5.18
High	LVSYDTVKDYAYIR		2	-0.29	2.04E-03	7.54E-04	5.12
High	SAITEFQNVQPTNEK		6	-0.71	2.04E-03	3.76E-04	5.1
High	VVSTTQNVAKPTTASSK		3	-0.02	2.04E-03	6.00E-05	5.07
High	SAITEFQNVQPTNEKMTDLQDTK	1xOxidation [M16]	1	0.32	2.04E-03	9.96E-04	5.05
High	TEAVASPTTTSEKAPETKPVANAVSVSNK		2	-0.52	2.04E-03	4.19E-06	4.88
High	DKTPATKPTKGEVESSSTTPTK		2	-1.24	2.04E-03	9.95E-06	4.8
High	TTKDVVQTSAGSSEAKDSAPLQK		2	-0.4	2.04E-03	1.39E-04	4.74
High	YMVMETTNDDYWK	1xOxidation [M2; M4]	2	-1.59	2.04E-03	1.08E-03	4.58
High	ANIKNTNDGHTQSQNNK		3	-0.21	2.04E-03	2.08E-04	4.53
High	APETKPVANAVSVSNK		2	-0.47	2.04E-03	2.81E-05	4.48
High	YDYTLMEFAQPIYNSADK	1xOxidation [M6]	1	1.73	2.04E-03	7.01E-04	4.47
High	TPATKPTKGEVESSSTTPTK		3	0.29	2.04E-03	3.05E-05	4.45
High	NTNDGHTQSQNNKNTQENK		1	-1.57	2.04E-03	2.14E-04	4.44
High	DDNKQLPSVEKENDASSESGK		1	-0.38	2.04E-03	4.06E-04	4.43
High	NTNDGHTQSQNNK		2	-0.48	2.04E-03	2.56E-03	4.38
High	YMVMETTNDDYWK	2xOxidation [M2; M4]	1	-0.47	2.04E-03	1.29E-03	4.37
High	YDYTLMEFAQPIYNSADK		1	-1.05	2.04E-03	1.70E-03	4.34
High	YMVMETTNDDYWK		1	0.22	2.04E-03	1.13E-03	4.28
High	KEATPATPSKPTPSPVEKESQK		2	0.42	2.04E-03	1.39E-04	4.2
High	QDSQKDDNKQLPSVEK		1	0.52	2.04E-03	4.67E-03	3.96
High	TTKDVVQTSAGSSEAK		2	0.24	2.04E-03	1.28E-03	3.92
High	DGTQQFYHYASSVKPAR		2	-0.6	2.04E-03	3.34E-03	3.89
High	ESQKQDSQKDDNK		9	-0.21	2.04E-03	4.24E-03	3.84
High	ATNNTYPILNQELR		5	0.81	2.04E-03	1.21E-03	3.79
High	TEAVASPTTTSEK		3	-0.59	2.04E-03	1.14E-02	3.78
High	DVVQTSAGSSEAK		2	-0.16	2.04E-03	1.98E-03	3.74
High	ANIKNTNDGHTQSQNNKNTQENK		1	0.24	2.04E-03	8.11E-03	3.74
High	KEATPATPSKPTPSPVEK		3	0.09	2.04E-03	7.21E-05	3.66
High	DDNKQLPSVEK		2	0.36	2.04E-03	1.68E-02	3.59
High	IVSSTHFNNKEEK		2	-0.5	2.04E-03	6.75E-03	3.55
High	EATPATPSKPTPSPVEKESQK		1	0.17	2.04E-03	2.99E-02	3.52
High	EATPATPSKPTPSPVEK		2	-0.12	2.04E-03	2.99E-04	3.39
High	TIDYDGQYHVR		2	-0.27	2.04E-03	5.55E-03	3.35
High	IQDKLPEKLK		1	-0.27	2.04E-03	2.98E-02	3.3
High	FKTEEDYKAEK		2	0.28	2.04E-03	1.73E-03	3.29
High	QVYELNKIQDK		2	-0.9	2.04E-03	4.26E-02	3.2
High	QLPSVEKENDASSESGK		1	-0.5	2.04E-03	1.48E-04	3.08
High	ENDASSESGKDKTPATKPTK		2	0.74	2.04E-03	4.33E-02	3.08
High	TEEDYKAEK		1	-0.83	2.04E-03	8.56E-02	3.01
High	KFEVYEGDKK		1	0.2	2.04E-03	6.21E-03	3
High	DFMVEGQR	1xOxidation [M3]	1	-1.32	2.04E-03	6.78E-02	3
High	MTDLQDTK		1	-0.32	2.04E-03	1.94E-02	2.94
High	MTDLQDTK	1xOxidation [M1]	1	0.91	2.04E-03	8.91E-02	2.9
High	GEVESSSTTPTK		2	-0.59	2.04E-03	6.10E-03	2.83
High	IVSSTHFNNK		2	-0.48	2.04E-03	1.84E-02	2.83
High	KFEVYEGDK		1	-0.52	2.04E-03	2.66E-02	2.72
High	DKDHSAPNSRPIDFEMK		1	-0.46	2.04E-03	5.50E-03	2.66
High	KALDEQVK		1	-0.23	2.04E-03	1.20E-01	2.65
High	TPATKPTK		1	0.01	2.04E-03	6.35E-02	2.58
High	FKTEEDYK		1	-0.12	2.04E-03	7.99E-02	2.57
High	ENDASSESGK		1	-0.11	2.04E-03	1.99E-02	2.54
High	VVSTTQNVAKPTTASSKTTK		1	-0.18	2.04E-03	1.15E-02	2.5
High	FEVYEGDK		1	0.4	2.04E-03	4.34E-02	2.49
High	SNKKEQQDNSAK		1	-0.61	2.04E-03	8.49E-02	2.49
High	IVDKEAFTK		1	0.3	2.04E-03	2.84E-02	2.48
High	VIFTDSKPEIELGLQSGQFWR		1	-1.14	2.04E-03	4.04E-02	2.37
High	DKDHSAPNSRPIDFEMK	1xOxidation [M16]	1	-0.5	2.04E-03	4.80E-02	2.35
High	TISKDAKNNTR		1	-0.1	2.04E-03	2.85E-02	2.31
High	DSAPLQK		3	1.19	2.04E-03	6.26E-02	2.21
High	TIIFPYVEGK		1	0.29	2.04E-03	1.49E-02	2.14
High	EVEAPTSETK		1	0.03	2.04E-03	4.98E-02	1.77
High	TLYDAIVK		1	0.92	2.04E-03	1.29E-02	1.71
High	QLPSVEKENDASSESGKDK		1	0.02	2.04E-03	4.80E-02	1.59
High	ENDASSESGKDK		2	0.08	2.04E-03	1.18E-01	1.31

Supplemental Table 1. Peptide sequences of anti-IsdB 1.5mAb-immunoprecipitated protein. The 70 unique IsdB peptide sequences identified in the mass spectroscopy study described in Figure 2 F are shown with their resolution confidence level, the oxidized amino acids, the number of peptide spectrum matches (#PSMs), the deviation of measured mass from the theoretical mass of the peptide (DeltaM (ppm)), the minimal false discovery rate (q value), the posterior error probability (PEP), and the cross correlation (Xcorr).
